# Impact of helminth infections during pregnancy on maternal and newborn Vitamin D and on birth outcomes

**DOI:** 10.1038/s41598-024-65232-9

**Published:** 2024-06-27

**Authors:** Sèyigbéna P. Déo-Gracias Berry, Yabo Josiane Honkpèhedji, Esther Ludwig, Saïdou Mahmoudou, Ulrich Fabien Prodjinotho, Rafiou Adamou, Odilon P. Nouatin, Bayode R. Adégbitè, Jean Claude Dejon-Agobe, Romuald Beh Mba, Moustapha Maloum, Anne Marie Mouima Nkoma, Jeannot Fréjus Zinsou, Adrian J. F. Luty, Meral Esen, Ayôla Akim Adégnika, Clarissa Prazeres da Costa

**Affiliations:** 1 Institute for Medical Microbiology, Immunology and Hygiene, TUM School of Medicine and Health, Technical University of Munich (TUM), Trogerstrasse 30, 81675 Munich, Germany; 2 Centre de Recherches Médicales de Lambaréné, Lambaréné, Gabon; 3 Center for Global Health, TUM School of Medicine and Health, Technical University of Munich (TUM), Munich, Germany; 4 Department of Parasitology, Leiden University Medical Centre, Leiden, The Netherlands; 5 Institut Für Tropenmedizin, Universität Tübingen, Tübingen, Germany; 6 Fondation Pour La Recherche Scientifique (FORS), Cotonou, Benin; 7 German Center for Infection Research (DZIF), Partner site Munich, Munich, Germany; 8 MERIT, IRD, Université Paris Cité, Paris, France; 9 German Center for Infection Research (DZIF), Partner site Tübingen, Tübingen, Germany; 10 Institut de Recherche Clinique du Bénin (IRCB), Abomey-Calavi, Benin

**Keywords:** Sub-Saharan Africa, Helminth infections, Maternal, Newborn vitamin D, Pregnancy outcomes, Parasitology, Nutritional supplements, Parasitic infection, Infectious diseases

## Abstract

Poor birth outcomes in low- and middle income countries are associated with maternal vitamin D deficiency and chronic helminth infections. Here, we investigated whether maternal *Schistosoma haematobium* affects maternal or cord vitamin D status as well as birth outcomes. In a prospective cross-sectional study of pregnant women conducted in Lambaréné, Gabon, we diagnosed maternal parasitic infections in blood, urine and stool. At delivery we measured vitamin D in maternal and cord blood. *S. haematobium*, soil-transmitted helminths, and microfilariae were found at prevalences of 30.2%, 13.0%, and 8.8%, respectively. Insufficient vitamin D and calcium levels were found in 28% and 15% of mothers, and in 11.5% and 1.5% of newborns. Mothers with adequate vitamin D had lower risk of low birthweight babies (aOR = 0.11, 95% CI 0.02–0.52, *p* = 0.01), whilst offspring of primipars had low cord vitamin D levels, and low vitamin D levels increased the risk of maternal inflammation. Maternal filariasis was associated with low calcium levels, but other helminth infections affected neither vitamin D nor calcium levels in either mothers or newborns. Healthy birth outcomes require maintenance of adequate vitamin D and calcium levels. Chronic maternal helminth infections do not disrupt those levels in a semi-rural setting in sub-Saharan Africa.

## Introduction

Pregnancy and fetal outcomes are influenced by multiple factors such as maternal genetics, environmental exposures, infections (e.g. helminths), nutrition, metabolic factors such as low Vitamin D (VitD)^1,2^. 25-hydroxyVitD (25(OH)D), also known as “calciferol”, is a fat-soluble secosterol present in some foods and is photosynthesized in vertebrate skin by ultraviolet B radiation^3,4^. It is mostly referred to as “Vitamin D” even though the active form of VitD is calcitriol or 1,25(OH)2D which is found in various tissues, including the liver, and during pregnancy, most importantly, in the placenta^3,5^. 25(OH)D is routinely measured to determine the VitD status of a person because of its long half-life (2–3 weeks) and since it has been shown to generally correlate well with the active form^6–8^. The main biological functions of calcitriol in general and during pregnancy are to promote calcium (Ca^2+^) absorption and to maintain adequate serum Ca^2+^ and phosphate levels to allow normal bone mineralization and growth of the fetus, and to maintain an immunosuppressive environment in the placenta, for example by attenuating inflammatory responses of macrophages and dendritic cells^9–16^.

The role of VitD on pregnancy outcomes and infant health status has recently attracted considerable interest, particularly in the context of the theory of “developmental origins of health and disease” (DOHaD), highlighting the key role of early life environmental conditions in shaping health and disease later in life^1,17,18^. Indeed, emerging evidence suggests that low maternal and fetal VitD are correlated and can aggravate allergic responses in offspring^1,19^. Furthermore, low VitD levels may lead to poor pregnancy outcomes including either skeletal (growth restriction) or non-skeletal health complications, pre-eclampsia, gestational diabetes, preterm birth, and low birthweight^20^. Alongside VitD, helminth parasites influence pregnancy outcomes especially in sub–Saharan Africa (SSA) where these chronic infections are common^21,22^. Indeed, maternal VitD deficiency and parasitic infections (e.g. with trematode schistosomes) have been associated with increased risks of maternal anemia and iron deficiency^20^. In addition, VitD deficiency is positively correlated with levels of inflammatory biomarkers such as white blood cells (WBC) or C-reactive protein (CRP)^23^. However, it has been shown that during pregnancy multiple factors such as stress and length of labour can also be associated with increased CRP levels^24^. We have recently shown that expression of genes related to steroid synthesis as well as to the VitD pathway were significantly lower in placental tissue from women in a helminth endemic low-income country when compared to a non-endemic high-income country^25^ indicating that more frequent exposure to pathogens such as helminths could impact VitD homeostasis and thus inflammation during pregnancy.

Chronic schistosomiasis during pregnancy, which affects approximately 40 million women of childbearing age, has indeed been shown to be a risk factor for poor pregnancy outcomes such as low birthweight^22,26,27^, miscarriage and anemia^21,27,28^, as well as VitD regulation and neonatal immune-modulation^29–31^ by as yet unknown pathways. We therefore aimed to investigate the influence of VitD levels and metabolic related factors during pregnancy in general and the impact of *Schistosoma haematobium (Sh)* and other parasitic infections on pregnancy and birth outcomes in a prospective study of Gabonese mothers and their newborns.

## Results

### Maternal and newborn biometric parameters, parasitic infection status, VitD, Ca^2+^ and CRP levels

**Figure 1:**
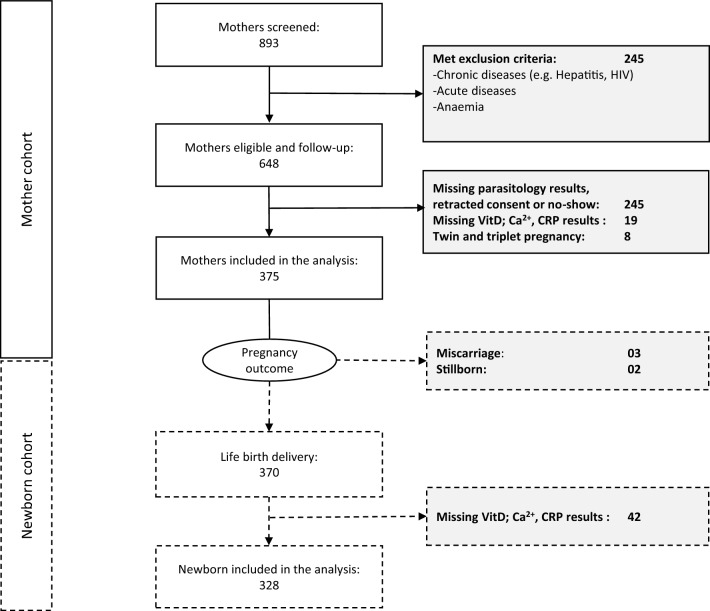
Flow chart of the participants during the study course. The mother cohort is shown as a solid line and the newborn cohort is shown as a broken line.

893 pregnant women or their legal representatives gave their informed consent. Of these, 648 pregnant women were eligible and followed up. Of these, 375 had VitD, Ca^2+^, CRP and parasitology results and were included in further analyses (Fig. 1). The median age (IQR) was 24 (20–30) years, there were 93 primigravidae (25%) and 276 multigravidae (74%), while 76 were primiparous (20%) and 190 multiparous (51%) (Suppl.Table 1). 370 infants were born alive and 328 were included in further analyses (Fig. 1). 176 (54%) were male. 275 (84%) had a birth weight appropriate to their gestational age (median [IQR] 3000 g [2717.5 to 3300]) while 14 (4.3%) had a low birth weight. The median length and head circumference (IQR) were 49 (48 to 51) cm and 33 (32 to 34) cm respectively (Suppl.Table 2).

**Figure 2:**
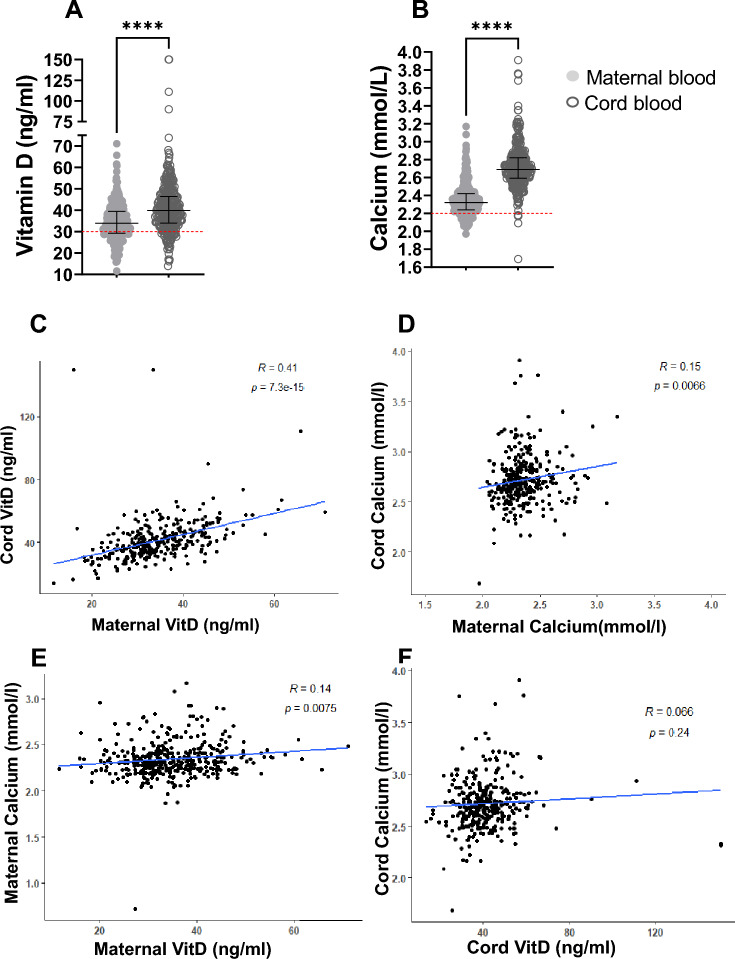
VitD and Ca^2+^ levels in maternal and cord blood at delivery. (**A**) VitD levels in maternal and cord blood (**B**) Ca^2+^ levels in in maternal and cord blood; red lines indicate VitD insufficiency threshold of 30 ng/mL and Ca^2+^ insufficiency threshold of 2,2 mmol/L; (**C**) correlation between maternal and cord VitD by Pearson’s correlation; (**D**) correlation between maternal and cord Ca^2+^ by Pearson’s correlation; (**E**) correlation between maternal Ca^2+^ and maternal VitD by Pearson’s correlation coefficient (**F**) correlation between cord Ca^2+^ and cord VitD by Pearson’s correlation; n (maternal) = 328; n (cord) = 328. Data are shown with median and interquartile range. *P* values are for Wilcoxon matched-pairs-Test. *P* value: * =  < 0,05; ** =  < 0,01; *** =  < 0,001; **** =  < 0,0001.

Comprehensive parasitological assessment was available for 289 (77.1%) of the 375 women and allocated to 171 NI, 60 *Sh*, 18 STH, 24 *Sh* + OI and 16 OI groups (Suppl.Fig. 2A;B). Thus, prevalence of parasitic infection at delivery was 30.2% for *Sh*, 13.0% for STH and, 8.8% for Mi. VitD levels were determined for 375 (100%). Among them, none were classified as VitD deficient, but 105 (28%) had insufficient whilst 270 (72%) had sufficient levels. In terms of serum Ca^2+^ concentration, 56 (15%) had insufficient, 284 (76%) adequate, whilst 35 (9.3%) had toxic levels. A total of 228 (61%) out of 375 reported taking oral Ca^2+^ and VitD supplements, the majority comprised of tablets (® CALCI D3-Denk) containing 1000 mg of Ca^2+^ and 800 IU VitD, while 144 (38%) reported not taking any such supplements during pregnancy. Interestingly, in our study group, only 162 women (43%) had normal CRP levels, while 182 (49%) had moderate and 31women (8.3%) had high CRP levels (Suppl.Table 1).

Cord blood analyses showed a substantially different picture in regards to VitD, Ca^2+^ and CRP levels: VitD concentrations in cord serum were deficient in only 5 (1.5%) newborns, insufficient in only 33 (10%), adequate in 287 (88%) and toxic in 3 (0.91%). Similarly, Ca^2+^ insufficiency in cord serum was detected only in 5 (1.5%) samples, 291 (89%) had a sufficient level and 32 (9.8%) had hypercalcemia. In line with this, 313 newborns (95%) had normal CRP levels and only 15 newborns (4.6%) had moderate CRP levels (Suppl.Table 2). These results clearly indicate that maternal nutrient insufficiency might be compensated by active transplacental transport which we further analyzed below (Fig. 2).

### Interdependence between maternal and/or newborn biometric parameters and maternal or cord VitD and Ca^2+^ levels

To investigate the relationship between materrnal and newborn VitD and Ca^2+^, we first used bivariate analyses to test for confounders by evaluating the relationship between the different maternal and newborn biometric and parasitological parameters and either VitD or Ca^2+^ level.

We found no associations of maternal VitD levels with the above parameters (Suppl.Table 3). However, when investigating whether any of the assessed maternal clinical parameters could influence her Ca^2+^ levels, we found that mothers aged under 18 as well as those over 30 had a significantly higher risk (*p* = 0.03) to have abnormal Ca^2+^ levels than mothers aged 18–29 (Suppl.Table 4).

With respect to cord VitD and Ca^2+^ levels, we furthermore found that primiparous mothers had a significantly higher risk to give birth to a child with low cord VitD levels (OR = 2.32, 95% CI 1.13–5.63, *p* = 0.03) (Suppl.Table 5) and that those born to mothers with filariasis had a significantly higher risk to have aberrant (low) cord Ca^2+^ levels (OR = 2.65, 95% CI 1.20–7.30, *p* = 0.03) (Suppl.Table 6). Taken together, parity and filariasis could influence cord VitD and Ca^2+^ respectively and needs to be considered when investigating mother–child axis in relation to infection.

### Association between birth outcomes and maternal or cord VitD and Ca^2+^ levels

We conducted multivariate regression analyses to assess the possible relationships between different birth outcomes and either maternal or cord VitD and Ca^2+^ levels. With respect to VitD levels, once adjusted, we found that mothers with adequate VitD levels had a significantly lower risk of giving birth to children with low birthweight (7.4% (aOR = 0.11, 95%CI: 0.02–0.52, *p* = 0.01)) when compared to newborns from mothers with low VitD levels (7.4%) (Table 1). With respect to Ca^2+^ level, no association was found (Table 2). However, the overall percentage of children with low birthweight was 5.2% and thus quite low in this cohort.

**Table 1:** Multivariate logistic regression assessing the association between maternal and cord VitD levels with newborn characteristics.

Variable	Maternal VitD (N = 375)					Cord VitD (N = 328)				
	Univariate			Multivariate		Univariate			Multivariate	
	Low	Adequate	*p*-value	aOR[95%CI]	*p*-value	Low	Adequate	*p*-value	aOR[95%CI]	*p*-value
Gende			0.331		0.401			0.223		0.076
Female	52 (50%)	119 (44%)		1		21 (55%)	129 (45%)		1	
Male	52 (50%)	149 (56%)		1.28 [0.72, 2.28]		17 (45%)	159 (55%)		2.26 [0.93, 5.77]	
Gestational age (weeks)			0.512		0.565			0.799		0.269
Normal [> = 37 weeks]	91 (88%)	218 (85%)		1		34 (89%)	241 (87%)		1	
Preterm [< 37 weeks]	13 (13%)	39 (15%)		1.27 [0.58, 3.03]		4 (11%)	37 (13%)		2.38 [0.62, 15.7]	
Birth weight (g)			0.283		**0.01**			0.667		0.123
Low [< 2500 g]	7 (7.4%)	11 (4.4%)		0.11 [0.02, 0.52]		2 (5.7%)	12 (4.4%)		0.23 [0.04, 1.88]	
Normal [> = 2500 g]	88 (93%)	239 (96%)		1		33 (94%)	258 (96%)		1	
Birth length (cm)			1		0.337			0.649		0.991
Abnormal [< 45; > 54 cm]	4 (4.0%)	11 (4.3%)		2.83 [0.49, 53.7]		2 (5.6%)	11 (3.9%)		5,074,924.78 [0.00, Inf]	
Normal[45-54 cm]	95 (96%)	246 (96%)		1	-	34 (94%)	269 (96%)		1	
Head circumference (cm)			0.571		0.985			0.16		0.973
Abnormal [< 30; > 38 cm]	3 (2.9%)	12 (4.6%)		5,916,152.36 [0.00, Inf]		3 (7.9%)	9 (3.2%)		0.96 [0.15, 18.9]	
Normal[30-38 cm]	99 (97%)	247 (95%)		1		35 (92%)	273 (97%)		1	

Data are presented as values or as proportions (%), core odd ratios (cOR), adjusted odd ratios (aOR), *p* value < 0.05 and 95% confidence intervals where indicated for VitD and adjusted by age group, parity and *Sh* infection status, blood for serum parameters was taken from maternal peripheral vein blood or from cord blood, respectively, missing data for each variable were not taken into analysis.

**Table 2:** Multivariate logistic regression investigating the association between maternal and cord Ca^2+^ levels with newborn characteristics.

Variable	Maternal calcium (N = 375)					Cord calcium (N = 328)				
	Univariate			Multivariate		Univariate			Multivariate	
	Normal	Abnormal	*p*-value	aOR[95%CI]	*p*-value	Normal	Abnormal	*p*-value	aOR[95%CI]	*p*-value
Gender			**0.023**		0.101			0.733		0.095
Female	139 (49%)	32 (36%)		1		132 (46%)	18 (49%)		1	
Male	143 (51%)	58 (64%)		1.68 [0.91, 3.17]		157 (54%)	19 (51%)		0.4 [0.13, 1.15]	
Gestational age (weeks)			0.224		0.26			0.786		0.872
Normal [> = 37 weeks]	238 (87%)	71 (82%)		1		246 (87%)	29 (85%)		1	
Preterm [< 37 weeks]	36 (13%)	16 (18%)		1.56 [0.70, 3.34]		36 (13%)	5 (15%)		0.87 [0.12, 3.95]	
Birth weight (g)			0.389		0.105			0.661		0.964
Low [< 2500 g]	12 (4.5%)	6 (7.4%)		3.36 [0.74, 15.2]		12 (4.4%)	2 (5.9%)		0.95 [0.04, 8.07]	
Normal [> = 2500 g]	252 (95%)	75 (93%)		1		259 (96%)	32 (94%)		1	
Birth length (cm)			0.21		0.408			0.649		0.991
Abnormal [< 45; > 54 cm]	9 (3.3%)	6 (7.1%)		1.87 [0.37, 8.08]		11 (3.9%)	2 (5.6%)		0 [NA]	
Normal[45-54 cm]	262 (97%)	79 (93%)		1		269 (96%)	34 (94%)		1	
Head circumference (cm)			1		0.512			1		0.994
Abnormal [< 30; > 38 cm]	12 (4.3%)	3 (3.6%)		0.49 [0.03, 2.90]		11 (3.9%)	1 (2.9%)		0 [NA]	
Normal[30-38 cm]	266 (96%)	80 (96%)		1		274 (96%)	34 (97%)		1	

Data are presented as values or as proportions (%), core odd ratios (cOR), adjusted odd ratios (aOR), *p* value < 0.05 and 95% confidence intervals where indicated for Ca^2+^ and adjusted by age group, parity and *Sh* infection status, blood for serum parameters was taken from maternal peripheral vein blood or from cord blood, respectively, missing data for each variable were not taken in analysis.

### Pairwise analysis of VitD and Ca^2+^ levels in maternal and cord blood at delivery and according to the season

In our cohort, VitD levels in pregnant women ranged from 12 to 66 ng/ml and in cord serum from 14 to 150 ng/ml with a median (95% CI) of 5.7 ng/ml (4.5 to 6.7) (Fig. 2A) relative to maternal values. Ca^2+^ levels ranged from 2.0 to 3.2 mmol/l in mothers, while in cord blood the range was 1.7 mmol/l to 3.7 mmol/l, with cord values compared to maternal values with a median (95% CI) of 0.37 mmol/l (0.35 to 0.40) (Fig. 2B). Compared to maternal serum and as mentioned above, cord blood contained significantly higher levels of VitD (41.4 ng/ml vs 34.6 ng/ml, *p* = 0.0001) and Ca^2+^ (2.7 vs 2.4 mmol/l, *p* = 0.0001). Paired analyses revealed that there was also a significant positive correlation between maternal and cord serum VitD (r = 0.41; *p* < 0.0001) and Ca^2+^ levels (r = 0.15; *p* = 0.0066) (Fig. 2C;D). In addition, the same result was observed between maternal Ca^2+^ and maternal VitD (r = 0.14; *p* = 0.0075). In term of R-square value (VitD: 17% and Ca^2+^: 2%) the percentage of association explained is respectively small or very small. However, there was no correlation between cord Ca^2+^ and cord VitD (r = 0.066; *p* = 0.24) (Fig. 2E;F). These results indicate that maternal deficiencies can be partially 'corrected' by active transport or sufficient placental production in 67% of neonates.

As VitD is influenced by the degree of exposure to sunlight^4^, we also studied the impact of the dry and rainy seasons on VitD levels in maternal and umbilical cord blood. In Gabon, there is a cooler dry season with less average sunshine: a short season from June to September. The rainy season, with more sunshine on average, extends from October to May^32,33^. VitD levels in cord blood were significantly higher than in maternal blood, regardless of the season (median (95% CI) 6.4 ng/ml (rainy season) (5.0 to 7.4); and 4.9 ng/ml (3.5 to 6.6)) (dry season) (*p* = 0.0001) and, overall, no seasonal differences were observed in maternal or cord blood. The same was true for Ca^2+^ levels (median (95% CI) 0.35 mmol/l (0.30 to 0.39) (rainy season); 0.39 mmol/l (0.36 to 0.44)) (*p* = 0.0001) (dry season) (Suppl.Fig. 1A;B).

### Effect of VitD and Ca^2+^ supplementation on maternal and cord blood levels

VitD and Ca^2+^ supplementation during pregnancy was reported in 61% of participants. But maternal VitD did not differ significantly for those with and without supplementation (*p* = 0.93). A majority in both groups (74.1% and 69.4%) had sufficient VitD levels, comparable to the overall 72% of women with sufficient VitD levels. In cord blood, maternal supplementation was not associated with altered VitD levels (*p* = 0.20) and cord blood levels were significantly higher than maternal VitD levels irrespective of maternal supplementation (*p* = 0.001) (Suppl.Table 7A;B). Concerning Ca^2+^ supplementation, identical sufficient blood Ca^2+^ levels were observed (*p* = 0.74) irrespective of maternal supplementation. Taken together, 76.3% and 75.7% of mothers, respectively, had sufficient Ca^2+^ levels. Ca^2+^ insufficiency was observed in 13.6% of mothers with and 16.0% of those without Ca^2+-^supplementation. Furthermore, regardless of maternal Ca^2+^ supplementation, there was no significant difference in newborn serum levels (78.1% vs 77.1%, with *p* = 0.75). Cord blood Ca^2+^ levels in both groups were significantly higher than in maternal serum at birth (*p* = 0.001) (Suppl.Table 8A;B). Thus, maternal oral VitD and Ca^2+^ supplementation had no impact on the respective serum levels of either mother or newborn and the data suggest an active regulation in fetal compartment.

### Effects of maternal helminth infection on maternal VitD and Ca^2+^ levels and on transplacental transfer

**Figure 3:**
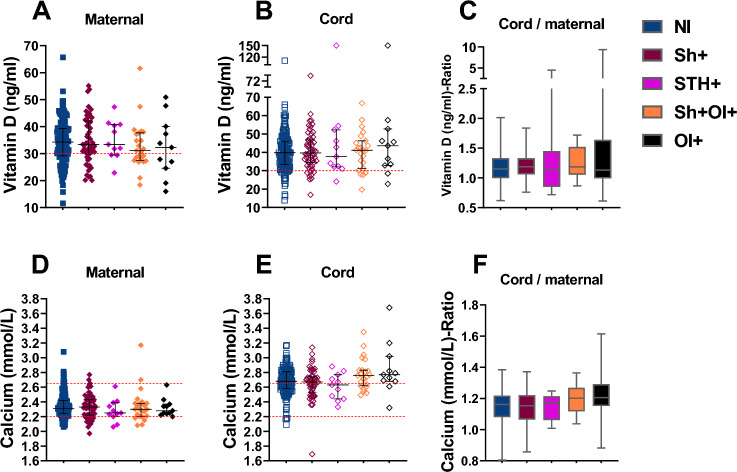
Effects of maternal helminth infection on maternal and newborn VitD and Ca^2+^ levels and the transplacental transfer. (**A**) maternal VitD according to the 5 main groups; (**B**) cord VitD according to the 5 main groups; (**C**) cord /maternal VitD ratio level within the 5 main groups; (**D**) maternal Ca^2+^ level within the 5 main groups; (**E**) cord Ca^2+^ level within the 5 main groups; (**F**) cord/maternal Ca^2+^ level within the 5 main groups. n (maternal N/I) = 137; n (maternal *Sh* +) = 55; n (maternal STH +) = 11; n (maternal *Sh* + OI +) = 23; (maternal OI +) = 11; n (cord NI) = 137; n (cord *Sh* +) = 55; n (cord STH +) = 11; n (cord *Sh* + OI) = 23; (cord OI +) = 11. VitD concentration Insufficiency: < 30 ng/mL, Ca^2+^ concentration Insufficiency: < 2,2 mmol/L. Data are shown with median and interquartile range. *P* values are for Mann–Whitney test. *P* value: * =  < 0,05; ** =  < 0,01; *** =  < 0,001; **** =  < 0,0001.

Despite overall sufficient VitD levels in the mothers, 28% remained below the recommended threshold of 30 ng/ml. To explore whether maternal helminth infection could play a role, especially in this group of mothers, we segregated the VitD and Ca^2+^ levels of mother–child pairs into five groups based on the parasitological diagnoses during enrolment as described above. No differences in maternal serum levels of either analyte were observed as a function of the infection status either alone or in combination (Fig. 3A,B,D,E). VitD and Ca^2+^ levels in cord blood were higher compared to maternal levels in all 5 NI, *Sh*, STH, *Sh* + OI and OI groups with fold changes of 1.15; 1.19; 1.14; 1.19; and 1.13 respectively for VitD and 1.16; 1.15; 1.17; 1.20; 1.20 respectively for Ca^2+^. This suggests an active feto-maternal transfer of Ca^2+^ and VitD or the sufficient placental production of the latter remains unperturbed and efficient irrespective of the presence of maternal parasitic infections (Fig. 3C;F).

### Impact of maternal inflammation on maternal and newborn C-reactive protein CRP level at delivery

**Figure 4:**
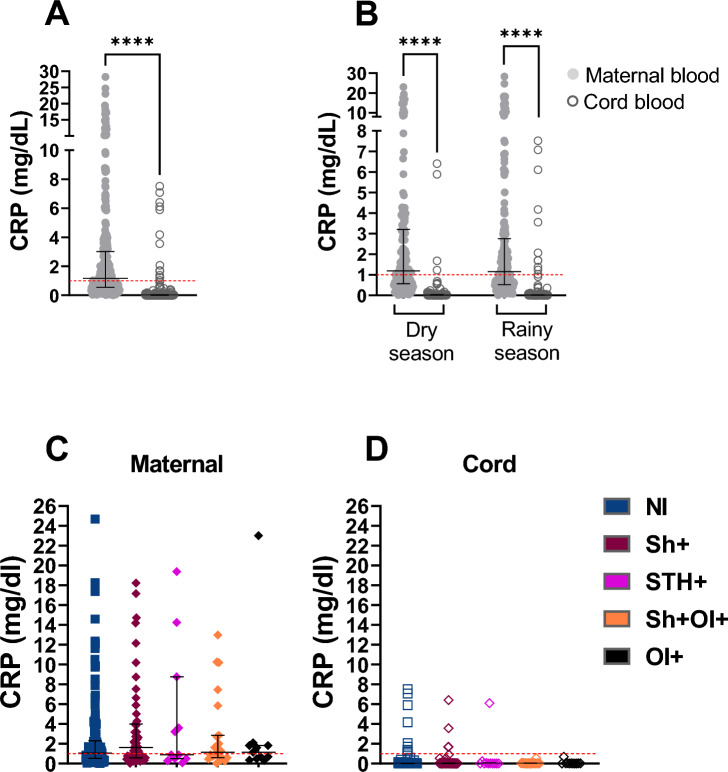
Impact of maternal infection on maternal and newborn C-reactive protein CRP level. (**A**) overall CRP level in all the cohort n (maternal) = 328; n (cord) = 328; (**B**) overall CRP level in the cohort according to the dry and rainy season n (maternal) = 328; n (cord) = 328; n (maternal N/I) = 137; n (maternal *Sh* +) = 55; n (maternal STH +) = 11; n (maternal *Sh* + OI) = 23; (maternal OI +) = 11; n (cord NI) = 137; n (cord *Sh* +) = 55; n (cord STH +) = 11; n (cord *Sh* + OI) = 23; (cord OI +) = 11; (**C**) maternal CRP level within the 5 main groups; (**D**) cord CRP level within the 5 main groups; CRP concentration : < 1 mg/dL. Data are shown with median and interquartile range. *P* values are for Wilcoxon matched-pairs-Test and Mann–Whitney test. *P* value: * =  < 0,05; ** =  < 0,01; *** =  < 0,001; **** =  < 0,0001.

To establish a possible effect of maternal inflammation, we measured CRP in maternal and cord blood. Elevated CRP levels were detected in only 8.3% of maternal sera at delivery (n = 31), with the majority (91.7%) showing normal or moderate levels (Suppl.Table 1). CRP was only detected in a relatively small number of newborns (n = 15), with no correlation with maternal CRP levels (Fig. 4A;B). Studying the latter group in more detail, we found no apparent explanation for the CRP levels observed in umbilical cord blood (e.g. LBW, low APGAR score, etc.). A Pearson correlation test was performed and showed that only physiological levels of CRP (< 1 mg/dl) were correlated between mother and child compared to abnormally high levels of maternal CRP (Suppl. Fig. 3A;B), indicating that there is no maternal transfer of CRP under these conditions, as CRP is not known to pass across the placenta. Furthermore, we found no association between CRP levels in maternal and cord samples as a function of maternal gravidity, parity or age (Suppl.Table 9). Interestingly, maternal parasitic infections did not influence maternal or neonatal CRP values either (Fig. 4C;D).

In addition to CRP, neutrophil and eosinophil counts were used to assess maternal inflammation. Bivariate (cOR = 0.41, 95% CI 0.19–0.85, *p* = 0.02) and multivariate (aOR = 0.25, 95% CI 0.08–0.70, *p* = 0.01) performed with VitD and CRP levels showed a statistically significant association between insufficient VitD in the cord and maternal CRP levels, in particular higher CRP levels (Suppl.Table 10), indicating the important role of VitD in regulating inflammation in this context. In addition, we found a trend towards the association of abnormal maternal eosinophil counts with insufficient maternal VitD (*p* = 0.06) and with insufficient cord VitD (*p* = 0.06) (Suppl.Table 11), whereas no association was found with maternal neutrophil counts and VitD levels in maternal and cord blood.

## Discussion

During pregnancy, the maternal immune system uses various immunomodulatory mechanisms to enable the foetus to survive despite being a xenograft. One of the factors that facilitates this immunomodulation is VitD, which is produced on both the maternal and fetal sides of the placenta and is considered vital for fetal development and placental homeostasis. VitD helps to maintain an immunosuppressive environment within the placenta^9,34,35^. However, helminths have also been shown to be powerful immunomodulators, and recent studies in both mice and humans have shown that maternal schistosomiasis can affect the immune system of the offspring^25,27,36^. As schistosomal infection has already been shown to reduce birth weight^22,26,37^, we sought to determine the impact of maternal helminth infection, and more specifically *Sh* infection, on pregnancy outcomes and on VitD and Ca^2+^ levels in maternal and cord blood, in a rural area of Gabon. We found that a significantly higher proportion of babies born to primiparous mothers and babies with low birth weight were born to mothers with low VitD levels. In addition, maternal and cord VitD appear to be influenced by both maternal CRP levels and eosinophil counts. However, most importantly, maternal *Sh* infection had no effect on maternal or neonatal VitD or Ca^2+^ levels, or on their respective transplacental transfer.

In accordance with the WHO guidelines^38^, we found that 72% of the pregnant women in our study cohort had sufficient serum levels of VitD, contrasting with results from high income countries (HIC) such as Germany where we recently found that only 34% of pregnant women had sufficient VitD levels^25^. Similar baseline levels of VitD were also reported in a recent study of rural Gambian pregnant women which increased as pregnancy progressed^32^. Interestingly, a recent systematic review and meta-analysis reported a surprisingly high prevalence up to 60% of VitD deficiencies in African countries. But the included studies were conducted mainly in urban and northern African settings highlighting that factors such as reduced exposure to sunlight, increasing urbanisation, differences in skin pigmentation, clothing style, and dietary habits need to be taken into consideration^37,39^.

We further identified maternal age as a factor potentially influencing maternal Ca^2+^ levels, findings aligning with the results of a study conducted in Indonesia where low serum Ca^2+^ levels were associated with the older mother group^40^. In addition, we found here that maternal filariasis negatively affects cord Ca^2+^ level. Maternal filariasis alters immune responses in newborns such that they are themselves more susceptible to filarial infection during early childhood^41–43^. However, the clinical or biological association between filarial infection and cord Ca^2+^ level remains unclear and needs further investigation.

A consistent and strong finding in this study was the feto-maternal alignment of VitD and Ca^2+^ levels which remained unperturbed by any other underlying factors such as parity or age. Also, in most newborns, maternal deficiencies were not reflected in the newborns hinting towards an active transfer of VitD and Ca^2+^ across the placenta to ensure healthy fetal growth^12^. These correlations likely also reflect the local production of VitD in the placenta which is regulated by peripheral VitD levels^44,45^ and likewise, several other studies have reported a positive correlation between maternal and umbilical cord blood VitD^25^. Nevertheless, some studies have also shown a correlation between maternal and cord blood VitD deficiency^4,46–48^. An explanation could be that these studies were conducted in context of nutritionally poor diets and chronic diseases such as HIV.

Our finding of an association between low maternal VitD levels and low birthweight is consistent with several studies that have reported similar findings^4,46,47,49–51^. These associations highlight the tight physiological link between VitD and Ca^2+^ levels and fetal growth and its potential regulation by factors such as nutrition, rural vs urban lifestyle. The primary factor governing VitD production is exposure to sunlight, and was thus shown previously to be influenced by seasonal changes and exposure to sunlight in different sub-Saharan African populations including pregnant women^39,52^. We however did not observe any seasonal changes which may appear surprising despite the fact that the rainy season in Lambaréné is associated with more prolonged periods of sunshine than the generally cloudy dry season.

According to WHO guidelines on antenatal care, oral VitD supplementation is not systematically recommended for all pregnant women to improve maternal and perinatal outcomes in populations with high sun exposure^53^. Our results show that VitD or Ca^2+^ supplementation (either self-reported or prescribed) had no impact on maternal or neonatal serum levels, as reported in a previous study comparing a cohort of German and Gabonese pregnant women^25^. However, other studies have shown that adequate VitD supplementation of the mother during pregnancy prevents premature delivery and influences the immune system of the newborn^54–56^.

This study also found that 8.3% of women had elevated CRP levels that could not be explained by underlying bacterial infection (see exclusion criteria), helminthic infection or other variables. Unfortunately, length of labour was not assessed in this study as a potential explanation for the elevated CRP levels. Instead, most children were negative or had physiological CRP levels. This lack of correlation suggests the existence of natural protective mechanisms that may mean that maternal CRP does not reflect local inflammation in the developing foetus. However, 15 newborns had non-physiological CRP levels and, in most cases, maternal CRP, which is known not to cross the placenta^57^, was absent. These 15 babies also showed no other signs of bacterial infection, distress or LBW, which does not allow us at present to explain this observation. Indeed, during pregnancy and labour, CRP is mainly used to identify underlying bacterial infection and/or to monitor prolonged labour^58,59^.

Accordingly, a study in schoolchildren in Cuba and in Cambodia found no substantial association between STH infections and maternal CRP, alpha-1 acid glycoprotein (AGP) or calprotectin as inflammation markers^60^. However, we observed that lower maternal and cord VitD levels are correlated with elevated maternal CRP levels and abnormal eosinophil counts, as has been reported in other studies on an older population in England; in pregnant women infected with COVID-19 or at risk of cardiovascular disease in Brazil and China respectively^61–63^. This may support the underlying concept that VitD has primarily an anti-inflammatory function, as demonstrated in many contexts^33^.

Finally, one of the main objectives of this study was to determine the influence of maternal helminth infection on VitD and Ca^2+^ levels, including maternal transfer to neonates, as we had previously found that signs of placental inflammation in Gabonese pregnant women could affect fetomaternal nutrient transport^25^. In the present study, we confirm active transport or sufficient placental production of VitD as previously described^64–70^. However, we found no influence of widespread maternal helminth infection on VitD and Ca^2+^ levels in maternal and cord blood, or on the active transport of either, which we consider an important public health finding. Indeed, a recent study reported no influence of *Plasmodium falciparum* or helminth infection on maternal vitamin D or birth outcomes^20^.

## Limitations

Size of study population was diminished by 38% due to either retracted consent, missing parasitology results or because of failure to show up for delivery. The duration of labour was not reported to assess high maternal CRP. STH prevalence was low. Also, our results cannot be generalised due to the exclusion of women with gestational diabetes, HIV, viral hepatitis and malaria.

In summary, our results show that in this mother–child cohort, only 28% and 15% of the mothers had insufficient VitD and Ca^2+^ levels which in most cases were resolved in the cord blood of the respective newborns indicating active and sufficient transport and local fetal production. Furthermore, maternal helminth infection during pregnancy had no impact on VitD levels and Ca^2+^ regulation in both mothers and their newborns except for maternal filariasis, which resulted in a higher risk for aberrant Ca^2+^ levels in newborns. Most importantly, we found that low maternal VitD levels were associated with low birthweight. Lastly, to the best of our knowledge, this is the first report on VitD in a large cohort of pregnant Gabonese women and their newborns, investigating its role in regulating maternal inflammation, and helminth infection-induced changes in its regulation during pregnancy.

## Materials and methods

### Study site

The study was conducted at CERMEL (Centre de Recherches Médicales de Lambaréne)^34^. Data and samples were collected from December 2018 to November 2020 in Lambaréné and the surrounding province of Moyen Ogooué situated at 77.85 km to the South of the Equator in the South-East of Gabon in central Africa. The rainfall is perennial except for the long dry season (from June to September) with a mean of 1216 mm per year^35^. The region is irrigated by the Ogooué River and its tributaries, with many ponds, lakes and streams constituting favourable conditions for fresh-water snail habitation. Water supply, fishing, household work, fetching water and playing are some activities which expose the local population to schistosomiasis^36^. Recently published data demonstrate that the prevalence in the area for *Sh* range from 30 to 75%^54,71^

### Study population and inclusion criteria

Pregnant women attending antenatal clinics (ANC) for routine and/or delivery visits at Hôpital Albert Schweitzer (ASH) and Centre Hospitalier Régional Georges Rawiri (CHRGR), two of the three health facilities located in the study area, were systematically invited to participate in the study. Volounteers willing to deliver and living in the area for at least one year before were eligible to participate in the study. Pregnant women with known chronic illnesses (diabetes, HIV, hepatitis B/C), acute illnesses (clinical malaria) likely to affect placental pathology and severe anaemia were excluded from the study.

### Samples size calculation

Adequacy of the sample size for determining the relationship between VitD metabolism and helminth carriage was evaluated using the formula for comparative studies with the specification of beta equal to 80% power and an alpha equal to 5% given on the possibility of loss of follow-up in clinical studies. Screening take place in known prevalence of *Sh* at 28% in Lambaréné region^54^. Therefore, a minimum of 180 women had to be included in the study.

### Study design and procedure

The study was designed as a prospective cross-sectional study. At baseline, demographics (age, sex and location) and anthropological (weight, height) data were collected. Axillary temperature was recorded. *Sh* infection, *P*. *falciparum* infection and soil-transmitted helminths (STH) status were assessed. EDTA blood, urine, and stool samples were collected from the pregnant women for parasitological diagnostics and basic biochemistry analysis at the first visit. The eligible participants were included and tested at baseline for the infection of interest, and the results were kept blinded for the member of research team in charge of their follow up. All participants were follow up in a similar manners. Study groups were determined based on the results, and allocated into five groups: Not-infected women (NI), positive for *Sh* alone (*Sh*), positive with soil-transmitted helminths (STH), positive for *Sh* and other infections (*Sh* + OI) and positive for other infections (OI) such as co-infections of STH with filarial diseases mainly *Loa loa* and *Mansonella perstans* and termed microfilaria (Mi). Pregnant women were asked to provide three urine sample for the diagnosis of urogenital schistosomiasis unless positive for the presence of *Sh* eggs in the first or the second sample. Percentages of neutrophils and eosinophils were documented with references: normal if neutrophils abs: [1.5−8.0] ×10^3^mm^3^ and eosinophils abs: [0.0–1.3] ×10^3^mm^3^ (Hematology Analyzer Yumizen H500).

Paired umbilical cord and maternal peripheral venous blood samples were collected at delivery. To avoid admixture of maternal and cord blood, the cord was cleaned and cord blood was obtained by direct needle aspiration avoiding squeezing. VitD, Ca^2+^ and CRP were detected in maternal and cord blood serum (see below). According to WHO guidelines^38^, an adequate level of VitD is defined as 30–100 ng/ml, whereas deficiency is defined as < 10 ng/ml, insufficiency as 10–29 ng/ml and toxicity as > 100 ng/ml. According to the Association of Perinatologist and the WHO guidelines^72,73^, the recommended range of serum Ca^2+^ is 2.2 to 2.6 mmol/l.^74^. However, in cord blood, a higher concentration is routinely observed and 3.00 mmol/l is considered excessive^75^. CRP, an acute phase protein and marker for inflammation, is considered normal if below 0.5 mg/dl whilst any levels above this threshold are considered abnormal and potential proof of inflammation^76^. However, in pregnancy, it is considered normal until 1 mg/dl, moderate between 1 and 10 mg/dl and higher over 10 mg/dl and these were the range used in this study^64^.

### Ethical consideration

The HelmVit study as a German-Gabonese collaborative project has been approved in Gabon by the institutional ethics committee of the Centre de Recherches Médicales de Lambaréné (CERMEL) (CEI-023/2018), in Germany by the university ethics committees of Munich (TUM) and Tübingen (UKT) (TUM 2016-349-S-KK ; UKT 515/2017BO1) . The study was registered on Clinical Trials.gov (registration number NCT04324853). All women gave written informed consent. For pregnant minors, signed consent was obtained from their legal representative or guardians in addition to their assent. The study was conducted in line with the Good Clinical Practice (GCP) principles of the International Conference on Harmonisation (ICH)^77^ and the Declaration of Helsinki^65^.

### Blood sample collection, processing, and preservation

All blood samples were collected in at least one 10 ml tube (two tubes were requested) containing NH_4_-Heparin (BD Vacutainer S-Monovette®) used for plasma separation by centrifugation with isolation and storage at − 80 for later analysis. Serum was isolated from maternal peripheral and cord blood using a 7 ml dry tube. The latter was centrifuged and the serum was immediately frozen in lightproof vials at − 80 °C until further analysis. The samples were stored at − 80 °C for long-term storage and shipped on dry ice to Munich, Germany.

### Parasitological examinations

As per CERMEL standard operating procedure and described elsewhere whole blood was used for the detection of microfilariae by Saponin concentration technique, and thick and thin blood smears were made for malaria detection with Giemsa staining and microscopical examination^78–81^. Urine filtration followed by microscopy was used for the diagnosis of urogenital schistosomiasis^53,55,82^. schistosome circulating antigen (circulating anodic antigen [CAA]) was measured utilizing an immunochromatography-based assay. This lateral flow (LF) test applies luminescent upconverting particles (UCP) to quantitatively measure CAA levels with a 2 pg/mL lower limit threshold^56^. Stool samples were analyzed by direct examination via Kato-Katz, the WHO gold standard for soil-transmitted helminth (STH) egg detection, Coproculture and Harada Mori culture techniques for detection of hookworm and *Strongyloides stercoralis* larvae and the saponin concentration technique for Filarasis^57^.

### Assessment of pregnancy outcomes

Pregnancy outcomes, including premature birth, stillbirth, normal delivery, miscarriage and infant anthropometric variables were evaluated immediately after delivery by midwife. Gestational age at delivery was defined in weeks based on the last date of menstruation and preterm delivery as less than 37 weeks of gestation.

### Biochemical analysis

VitD was assessed using the Diasorin assay (Diasorin, Stillwater, MN, USA). Ca^2+^ values were measured on a Roche/Hitachi Cobas c501 analyzer. For CRP, the Tina-quant® C-Reactive Protein assay (Cobas Roche diagnostic kit) was used. Both assays were carried out according to the manufacturer’s instructions at the Institute for Clinical Chemistry and Pathobiochemistry at the Klinikum Rechts der Isar of TUM.

### Statistical analysis

Data were captured on the patient report form (PRF), entered in RedCap data collection platform and transferred to R statistical software (version 4.2.2), and PRISM® 9.10 (GraphPad Software Inc., San Diego, CA, USA). Continuous data were tested for normality using the Kolmogorov–Smirnov test. If not normally distributed, continuous variables were expressed as medians with interquartile ranges (IQR) and significance was calculated using the Wilcoxon Rank Sum test or by the mean and standard deviation (SD). The chi-squared test was used to compare proportions. For the bivariate and multivatriate analysis, we categorized all VitD values < 30 ng/ml as low and > 30 ng/ml as adequate. Ca^2+^ was categorized as abnormal if < 2.2 and/or > 2.6 mmol/l in maternal blood or > 3 mmol/l in Cord blood and normal between 2.2 and 26 mmol/l.For *Sh* infection, participant was considered as infected if positive for the presence of at least one egg in urine or positive for CAA*.* For *A. lumbricoides*, *T. trichiura*, and Hookworm infection, participant was considered as infected if positive for the presence of at least one egg in stool sample. For Hookworm and *S. stercoralis* infection participant was considered as infected if at least one larvae was found in stool sample. For Filarasis, participant was considered as positive if at least one microfilariae was found in the blood. We used bivariate analyses to test for confounders by evaluating the relationship between VitD or Ca^2+^ levels as the main variables and each exploratory variable. A difference of 25% or more in the estimated measure of association before and after adjustment was used to define confounding factors (age group, parity and *Sh* infection status). Multivariate stepwise logistic regression analyses were performed to investigate a possible association between levels of VitD, Ca2+ , CRP, neutrophils and eosinophils, and maternal and/or neonatal variables; odds ratio (OR) and confidence intervals were calculated using R's "compare groups" function. Factors used for adjustment in the multivariate analyses were age group, parity and *Sh* infection status. Correlations between maternal and cord VitD, Ca^2+^, CRP were assessed using Pearson’s coefficient. The significance level for all calculations was set at *p* < 0.05.

## Supplementary Information

Supplementary Information.

## Data Availability

All data generated or analysed during this study are included in this published article and its supplementary information files.
