# Publisher Correction: Impact of helminth infections during pregnancy on maternal and newborn Vitamin D and on birth outcomes

**DOI:** 10.1038/s41598-024-70356-z

**Published:** 2024-08-22

**Authors:** Sèyigbéna P. Déo‑Gracias Berry, Yabo Josiane Honkpèhedji, Esther Ludwig, Saïdou Mahmoudou, Ulrich Fabien Prodjinotho, Rafiou Adamou, Odilon P. Nouatin, Bayode R. Adégbitè, Jean Claude Dejon‑Agobe, Romuald Beh Mba, Moustapha Maloum, Anne Marie Mouima Nkoma, Jeannot Fréjus Zinsou, Adrian J. F. Luty, Meral Esen, Ayôla Akim Adégnika, Clarissa Prazeres da Costa

**Affiliations:** 1 Institute for Medical Microbiology, Immunology and Hygiene, TUM School of Medicine and Health, Technical University of Munich (TUM), Trogerstrasse 30, 81675 Munich, Germany; 2 Centre de Recherches Médicales de Lambaréné, Lambaréné, Gabon; 3 Center for Global Health, TUM School of Medicine and Health, Technical University of Munich (TUM), Munich, Germany; 4 Department of Parasitology, Leiden University Medical Centre, Leiden, The Netherlands; 5 Institut Für Tropenmedizin, Universität Tübingen, Tübingen, Germany; 6 Fondation Pour La Recherche Scientifique (FORS), Cotonou, Benin; 7 German Center for Infection Research (DZIF), Partner site Munich, Munich, Germany; 8 MERIT, IRD, Université Paris Cité, Paris, France; 9 German Center for Infection Research (DZIF), Partner site Tübingen, Tübingen, Germany; 10 Institut de Recherche Clinique du Bénin (IRCB), Abomey-Calavi, Benin

Correction to: *Scientific Reports* 10.1038/s41598-024-65232-9, published online 27 June 2024

The Acknowledgements section in the original version of this Article was incomplete.

“We are grateful to all the pregnant women who participated in the study. We thank all the CERMEL staff (especially the nurses and the parasitology unit) for their exceptional help, and we are indebted to the midwives of the GR Regional Hospital and those of the HAS, to all the nurses, field workers and doctors involved in this project and to the CERMEL staff. Thanks to Dominik Stelzle for his involvement in the data cleansing process. This work was supported and funded by DFG CO 1469/14-1, DFG ES 153/12-1.”

now reads:

“We are grateful to all the pregnant women who participated in the study. We thank all the CERMEL staff (especially the nurses and the parasitology unit) for their exceptional help, and we are indebted to the midwives of the GR Regional Hospital and those of the HAS, to all the nurses, field workers and doctors involved in this project and to the CERMEL staff. Thanks to Dominik Stelzle for his involvement in the data cleansing process. This work was supported and funded by DFG CO 1469/14-1, DFG ES 153/12-1 RTG2668 (Project A06, Project-ID: 435874434) Deutsche Forschungsgemeinschaft.”

The original Article has been corrected.

