# Supplementary material for: Impact of helminth infections during pregnancy on maternal and newborn Vitamin D and on birth outcomes
Source: Sci Rep. 2024 Jun 27;14:14845. doi: 10.1038/s41598-024-65232-9 (PMC11211496; doi:10.1038/s41598-024-65232-9)
Supplement: Supplementary file 1 — Supplementary Information. [file 41598_2024_65232_MOESM1_ESM.pdf]

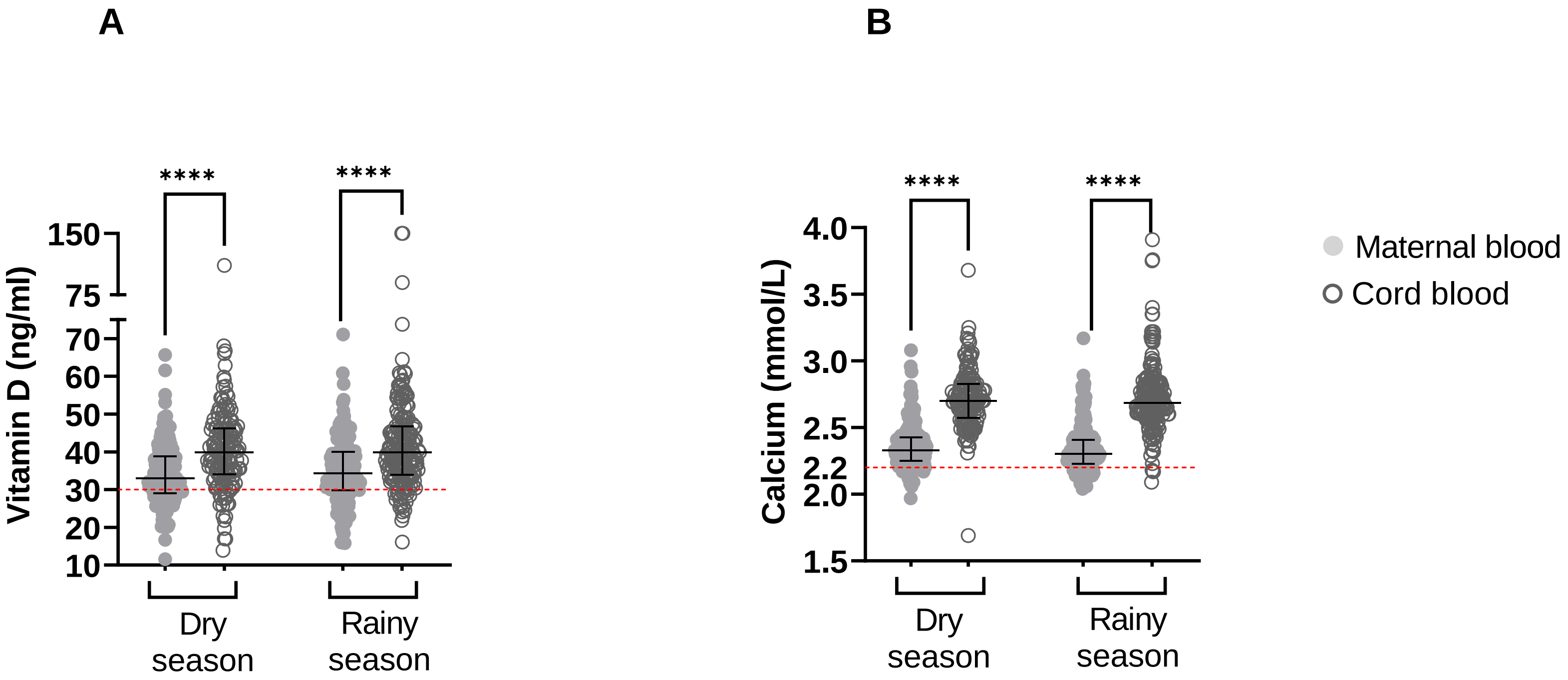


## Suppl. Figure 1: Seasonal VitD and $Ca^{2+}$ levels

(A) overall VitD level in all the cohort according to the dry and rainy season; (B) overall $Ca^{2+}$ level in the cohort according to the dry and rainy season; n (maternal) = 328; n (cord) = 328 VitD concentration Insufficiency: < 30ng/mL, $Ca^{2+}$ concentration Insufficiency: < 2,2mmol/L. Data are shown with median and interquartile range. P values are for Wilcoxon matched-pairs-Test. P value: * = < 0,05; ** = < 0,01; *** = < 0,001; **** = < 0,0001.

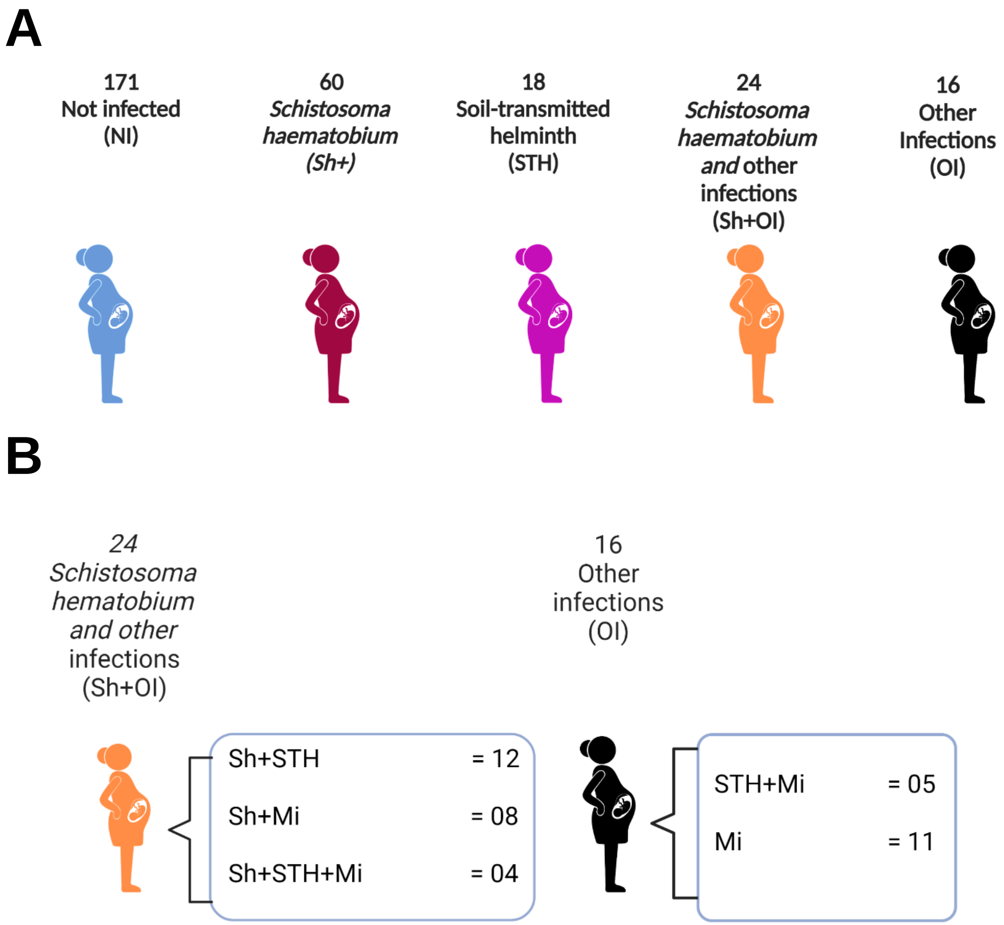


**Suppl. Figure 2: Parasitological infection status of the mothers at recruitment**

(A) Illustration describing the parasitological status in 5 major groups: Not infected (NI) =171 women, *S. haematobium* (*Sh)* infected group (*Sh+*) = 60 women, soil-transmitted-helminths group (STH+) = 18 women, *Schistosoma haematobium and* coinfected group (*Sh*+OI) = 24 women and group with other infections (OI) = 16 women. (B) detailed shutdown within the (*Sh*+OI) group: *Schistosoma haematobium* (*Sh+*) and soil-transmitted-helminths (STH+) = 12 women; *Schistosoma haematobium* (*Sh+*), soil-transmitted-helminths (STH+) and microfilaria (Mi+) = 4 women; *Schistosoma haematobium* (*Sh+*) and microfilaria (Mi+) = 08 women. The OI group: 05 women soil-transmitted-helminths (STH+) and microfilaria (Mi+) infections; 11 women microfilaria (Mi+) infections.

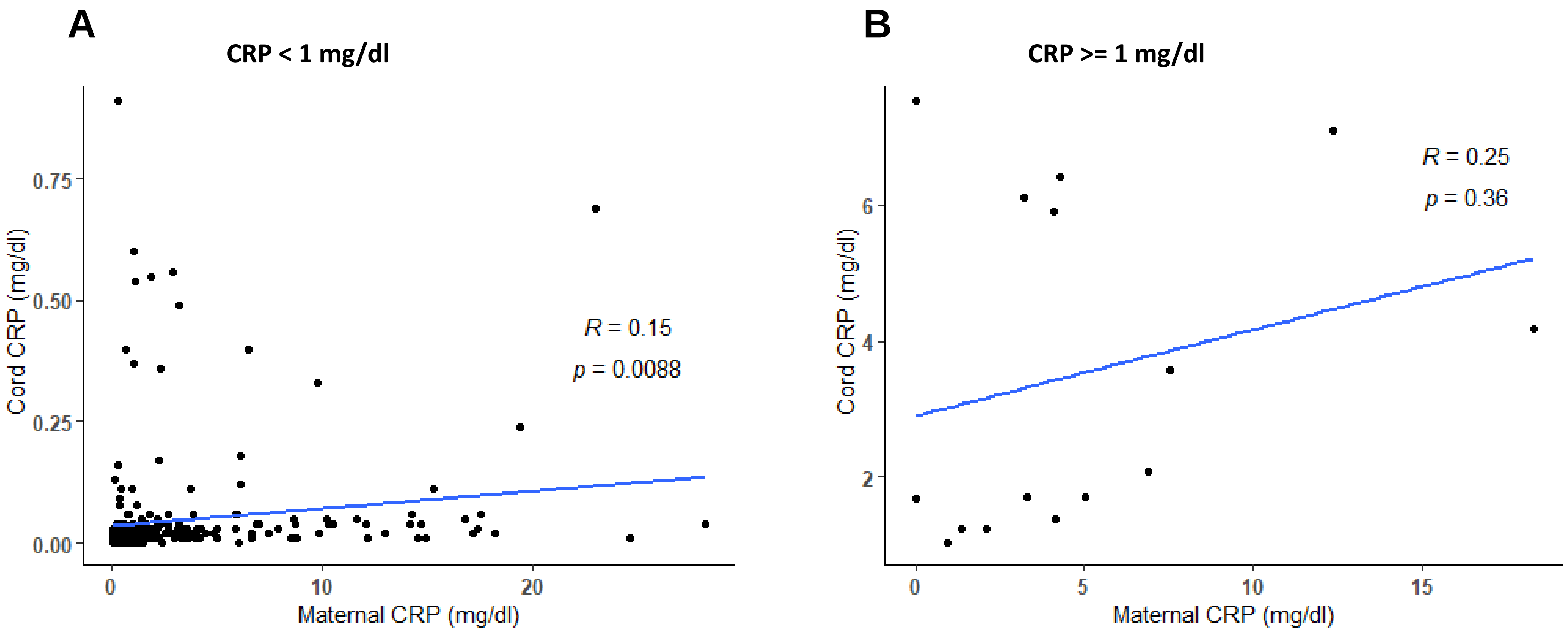


**Suppl. Figure 3: Correlation between maternal and cord CRP**

Data are shown as correlation between maternal and cord CRP by Pearson's correlation; (A) CRP cord < 1 mg/dl; (B) CRP cord >= 1 mg/dl.

Suppl Table 1: Maternal characteristics at birth (N = 375)

| **Variable** | | **N** | **%** |
|---|---|---|---|
| Maternal age group (years) | 13 - 17 | 29 | 7.7 |
| | 18 - 29 | 241 | 64 |
| | 30 - 45 | 105 | 28 |
| | Median (IQR) | 24 (20 to 30) | - |
| Gravidity | Primigravidae | 93 | 25 |
| | Multigravidae | 276 | 74 |
| Parity | Primiparity | 76 | 20 |
| | Multiparity | 190 | 51 |
| Season | Dry | 157 | 42 |
| | Rainy | 218 | 58 |
| Maternal VitD | Insufficient (10-29 ng/ml) | 105 | 28 |
| | Sufficient (30 -100 ng/ml) | 270 | 72 |
| Maternal calcium | Insufficient (< 2.2 mmol/l) | 56 | 15 |
| | Sufficient (2.2-2.6 mmol/l) | 284 | 76 |
| | Toxic (> 2.6 mmol/l) | 35 | 9.3 |
| Maternal CRP (mg/dl) | Normal[<1mg/dl] | 162 | 43 |
| | Moderate [1-10 mg/dl] | 182 | 49 |
| | Higher [>10 mg/dl] | 31 | 8.3 |
| Calcium supplementation | Yes | 228 | 61 |
| | No | 144 | 38 |
| Vitamin D supplementation | Yes | 228 | 61 |
| | No | 144 | 38 |
| Maternal VitD | Low (< 30 ng/ml) | 105 | 28 |
| | Adequate (> 30 ng/ml) | 270 | 72 |
| Maternal calcium | Abnormal | 91 | 24 |
| | Normal | 284 | 76 |

Data are presented as median with IQR (n) for maternal age, values or as proportions (%) and 95% confidence intervals where indicated, missing data for each variable were not taken into analysis.

## Suppl Table 1. Overall maternal characteristics at birth

Data are presented as median with IQR (n) for maternal age, values or as proportions (%) and 95% confidence intervals where indicated; Khi2 test was performed for the percentage comparison; blood for serum parameters was taken from maternal venous blood, missing data for each variable were not taken into the analysis.

Suppl Table 2: Newborn characteristics at birth (N = 328)

| Variable | | N | % |
|---|---|---|---|
| Sex | Female | 150 | 46 |
| | Male | 176 | 54 |
| Gestational age (weeks) | Preterm [< 37 weeks] | 41 | 12 |
| | Normal [>= 37 weeks] | 275 | 84 |
| | Median (IQR) | 39 (38 to 40) | - |
| Birth weight (g) | Low [< 2500g] | 14 | 4.3 |
| | Normal [>= 2500g] | 291 | 89 |
| | Median (IQR) | 3000 (2717.5 to 3300) | - |
| Birth length (cm) | Abnormal [<45; >54cm] | 13 | 4 |
| | Normal[45-54cm] | 303 | 92 |
| | Median (IQR) | 49 (48 to 51) | - |
| Head circumference (cm) | Abnormal [<30; >38cm] | 12 | 3.7 |
| | Normal[30-38cm] | 308 | 94 |
| | Median (IQR) | 33 (32 to 34) | - |
| Cord serum VitD | Deficient (< 10 ng/ml) | 5 | 1.5 |
| | Insufficient (10-29 ng/ml) | 33 | 10 |
| | Sufficient (30 -100 ng/ml) | 287 | 88 |
| | Toxic (> 100 ng/ml) | 3 | 0.91 |
| Cord serum calcium | Insufficient (< 2.2 mmol/l) | 5 | 1.5 |
| | Sufficient (2.2-3 mmol/l) | 291 | 89 |
| | Toxic (> 3 mmol/l) | 32 | 9.8 |
| Cord serum CRP (mg/dl) | Normal[<1mg/dl] | 313 | 95 |
| | Moderate [1-10 mg/dl] | 15 | 4.6 |
| Cord serum VitD | Low (< 30 ng/ml) | 38 | 12 |
| | Adequate (> 30 ng/ml) | 290 | 88 |
| Cord serum calcium | Normal | 291 | 89 |
| | Abnormal | 37 | 11 |

Data are presented as median with IQR (n) for gestational age at birth, birth weight, birth length, head circumference, values or as proportions (%) and 95% confidence intervals where indicated, missing data for each variable were not taken into analysis

## Suppl Table 2. Overall newborn characteristics at birth

Data are presented as median with IQR (n) for gestational age at birth, birth weight, birth length, head circumference, values or as proportions (%) and 95% confidence intervals where indicated; Khi2 test was performed for the percentage comparison; serum parameters were determined from cord blood, missing data for each variable were not taken into the analysis.

Suppl Table 3: Association between mother's clinical characteristics and the maternal VitD level

| Variable | | Total | Adequate (> 30 ng/ml) | Low (< 30 ng/ml) | p. overall | OR [95%CI] | p. ratio |
|---|---|---|---|---|---|---|---|
| Maternal age group (years) | | | | | 0.490 | | |
| | 13 - 17 | 29 (7.73%) | 23 (8.52%) | 6 (5.71%) | | 1 | |
| | 18 - 29 | 241 (64.3%) | 175 (64.8%) | 66 (62.9%) | | 1.23 [0.55;3.41] | 0.459 |
| | 30 - 45 | 105 (28.0%) | 72 (26.7%) | 33 (31.4%) | | 1.49 [0.64;4.36] | 0.270 |
| Gravidity | | | | | 0.956 | | |
| | Multigravidae | 276 (74.8%) | 199 (74.5%) | 77 (75.5%) | | 1 | |
| | Primigravidae | 93 (25.2%) | 68 (25.5%) | 25 (24.5%) | | 0.92 [0.57;1.62] | 0.858 |
| Parity | | | | | 0.913 | | |
| | Multiparity | 190 (71.4%) | 137 (71.0%) | 53 (72.6%) | | 1 | |
| | Primiparity | 76 (28.6%) | 56 (29.0%) | 20 (27.4%) | | 0.89 [0.51;1.69] | 0.804 |
| Maternal Hemoglobin | | | | | 0.963 | | |
| | HB < 11 | 214 (69.3%) | 151 (68.9%) | 63 (70.0%) | | 1 | |
| | HB >= 11 | 95 (30.7%) | 68 (31.1%) | 27 (30.0%) | | 0.92 [0.56;1.63] | 0.863 |
| Maternal CRP | | | | | 0.741 | | |
| | Normal[<1mg/dl] | 162 (43.2%) | 115 (42.6%) | 47 (44.8%) | | 1 | |
| | Moderate [1-10 mg/dl] | 182 (48.5%) | 134 (49.6%) | 48 (45.7%) | | 0.85 [0.55;1.40] | 0.587 |
| | Higher [>10 mg/dl] | 31 (8.27%) | 21 (7.78%) | 10 (9.52%) | | 1.09 [0.53;2.67] | 0.711 |
| Maternal Neutrophils | | | | | 0.727 | | |
| | Abnormal | 23 (8.16%) | 15 (7.54%) | 8 (9.64%) | | 1 | |
| | Normal | 259 (91.8%) | 184 (92.5%) | 75 (90.4%) | | 0.68 [0.31;1.80] | 0.557 |
| Maternal Eosinophils | | | | | 1.000 | | |
| | Abnormal | 14 (4.95%) | 10 (4.98%) | 4 (4.88%) | | 1 | |
| | Normal | 269 (95.1%) | 191 (95.0%) | 78 (95.1%) | | 0.81 [0.31;2.98] | 0.998 |
| *S. haematobium* | | | | | 0.815 | | |
| | Non-infected | 233 (68.3%) | 166 (67.8%) | 67 (69.8%) | | 1 | |
| | Positive | 108 (31.7%) | 79 (32.2%) | 29 (30.2%) | | 0.88 [0.55;1.52] | 0.723 |
| STH | | | | | 0.249 | | |
| | Non-infected | 233 (86.9%) | 166 (88.8%) | 67 (82.7%) | | 1 | |
| | Positive | 35 (13.1%) | 21 (11.2%) | 14 (17.3%) | | 1.55 [0.81;3.43] | 0.189 |
| Microfilaria | | | | | 1.000 | | |
| | Non-infected | 233 (87.9%) | 166 (87.8%) | 67 (88.2%) | | 1 | |
| | Positive | 32 (12.1%) | 23 (12.2%) | 9 (11.8%) | | 0.92 [0.45;2.23] | 0.959 |
| Any Infection | | | | | 0.765 | | |
| | Non-infected | 233 (62.1%) | 166 (61.5%) | 67 (63.8%) | | 1 | |
| | Positive | 142 (37.9%) | 104 (38.5%) | 38 (36.2%) | | 0.88 [0.57;1.45] | 0.681 |

Data are presented as Odd ratios (OR) and 95% confidence intervals for VitD, *P* value significative < 0,05, blood for serum parameters was taken from maternal peripheral vein blood or from cord blood, respectively, missing data for each variable were not taken into analysis

**Suppl Table 3: Association between mother's clinical characteristics and the maternal VitD level**

Data are presented as Odd ratios (OR) and 95% confidence intervals for VitD, *P* value significative < 0,05, blood for serum parameters was taken from maternal peripheral vein blood or from cord blood, respectively, missing data for each variable were not taken into analysis

Suppl Table 4: Association between mother's clinical characteristics and maternal $Ca^{2+}$ level

| Variable | | Total | Normal | Abnormal | p. overall | OR [95%CI] | p. ratio |
|---|---|---|---|---|---|---|---|
| Maternal age group (years) | | | | | **0.031** | | |
| | 13 -17 | 29 (7.73%) | 20 (7.04%) | 9 (9.89%) | | 1 | |
| | 18-29 | 241 (64.3%) | 193 (68.0%) | 48 (52.7%) | | 0.49 [0.24;1.24] | 0.184 |
| | 30 - 45 | 105 (28.0%) | 71 (25.0%) | 34 (37.4%) | | 0.94 [0.44;2.49] | 0.905 |
| Gravidity | | | | | 0.959 | | 0.859 |
| | Multigravidae | 276 (74.8%) | 208 (74.6%) | 68 (75.6%) | | 1 | |
| | Primigravidae | 93 (25.2%) | 71 (25.4%) | 22 (24.4%) | | 0.92 [0.55;1.65] | |
| Parity | | | | | 0.425 | | 0.345 |
| | Multiparity | 190 (71.4%) | 142 (70.0%) | 48 (76.2%) | | 1 | |
| | Primiparity | 76 (28.6%) | 61 (30.0%) | 15 (23.8%) | | 0.70 [0.39;1.41] | |
| Maternal Hemoglobin | | | | | 0.481 | | 0.404 |
| | HB < 11 | 214 (69.3%) | 157 (68.0%) | 57 (73.1%) | | 1 | |
| | HB >= 11 | 95 (30.7%) | 74 (32.0%) | 21 (26.9%) | | 0.76 [0.45;1.39] | |
| Maternal CRP | | | | | **0.010** | | |
| | Normal [<1mg/dl] | 162 (43.2%) | 130 (45.8%) | 32 (35.2%) | | 1 | |
| | Moderate [1-10 mg/dl] | 182 (48.5%) | 137 (48.2%) | 45 (49.5%) | | 1.28 [0.80;2.21] | 0.273 |
| | High [>10 mg/dl] | 31 (8.27%) | 17 (5.99%) | 14 (15.4%) | | 3.06 [1.50;7.37] | **0.005** |
| Maternal Neutrophils | | | | | 1.000 | | 0.798 |
| | Abnormal | 23 (8.16%) | 17 (7.94%) | 6 (8.82%) | | 1 | |
| | Normal | 259 (91.8%) | 197 (92.1%) | 62 (91.2%) | | 0.76 [0.33;2.19] | |
| Maternal Eosinophils | | | | | 0.340 | | 0.332 |
| | Abnormal | 14 (4.95%) | 9 (4.21%) | 5 (7.25%) | | 1 | |
| | Normal | 269 (95.1%) | 205 (95.8%) | 64 (92.8%) | | 0.47 [0.18;1.61] | |
| *S. haematobium* | | | | | 0.548 | | 0.464 |
| | Non-infected | 233 (68.3%) | 179 (69.4%) | 54 (65.1%) | | 1 | |
| | Positive | 108 (31.7%) | 79 (30.6%) | 29 (34.9%) | | 1.18 [0.73;2.06] | |
| STH | | | | | 1.000 | | 0.989 |
| | Non-infected | 233 (86.9%) | 179 (86.9%) | 54 (87.1%) | | 1 | |
| | Positive | 35 (13.1%) | 27 (13.1%) | 8 (12.9%) | | 0.93 [0.45;2.33] | |
| Microfilaria | | | | | 0.995 | | 0.803 |
| | Non-infected | 233 (87.9%) | 179 (88.2%) | 54 (87.1%) | | 1 | |
| | Positive | 32 (12.1%) | 24 (11.8%) | 8 (12.9%) | | 1.04 [0.49;2.64] | |
| Any Infection | | | | | 0.612 | | 0.529 |
| | Non-infected | 233 (62.1%) | 179 (63.0%) | 54 (59.3%) | | 1 | |
| | Positive | 142 (37.9%) | 105 (37.0%) | 37 (40.7%) | | 1.14 [0.72;1.89] | |

Data are presented as Odd ratios (OR) and 95% confidence intervals for $Ca^{2+}$, *P* value significance < 0,05, blood for serum parameters was taken from maternal peripheral vein blood or from cord blood, respectively, missing data for each variable were not taken into analysis

**Suppl Table 4. Effects of maternal biometric parameters on maternal $Ca^{2+}$ level:** Data are presented as Odd ratios (OR) and 95% confidence intervals for $Ca^{2+}$, *P* value significative < 0,05, blood for serum parameters was taken from maternal peripheral vein blood or from cord blood, respectively, missing data for each variable were not taken into the analysis.

Suppl Table 5: Association of maternal and newborn characteristics with cord VitD level

| Variable | | Total | Adequate | Low | p. overall | OR [95%CI] | p. ratio |
|---|---|---|---|---|---|---|---|
| Gender | | | | | 0.296 | | 0.231 |
| | Female | 150 (46.0%) | 129 (44.8%) | 21 (55.3%) | | 1 | - |
| | Male | 176 (54.0%) | 159 (55.2%) | 17 (44.7%) | | 0.62 [0.34;1.29] | |
| Birth weight (g) | | | | | 0.667 | | 0.701 |
| | Low [< 2500g] | 14 (4.59%) | 12 (4.44%) | 2 (5.71%) | | 1 | - |
| | Normal [>= 2500g] | 291 (95.4%) | 258 (95.6%) | 33 (94.3%) | | 0.51 [0.16;2.64] | |
| Birth length (cm) | | | | | 0.649 | | 0.623 |
| | Abnormal [<45; >54cm] | 13 (4.11%) | 11 (3.93%) | 2 (5.56%) | | 1 | - |
| | Normal[45-54cm] | 303 (95.9%) | 269 (96.1%) | 34 (94.4%) | | 0.46 [0.14;2.42] | |
| Head circumference (cm) | | | | | 0.160 | | 0.201 |
| | Abnormal [<30; >38cm] | 12 (3.75%) | 9 (3.19%) | 3 (7.89%) | | 1 | - |
| | Normal[30-38cm] | 308 (96.2%) | 273 (96.8%) | 35 (92.1%) | | 0.29 [0.10;1.26] | |
| Gestational age (weeks) | | | | | 0.799 | | 0.670 |
| | Normal [>= 37 weeks] | 275 (87.0%) | 241 (86.7%) | 34 (89.5%) | | 1 | - |
| | Preterm [< 37 weeks] | 41 (13.0%) | 37 (13.3%) | 4 (10.5%) | | 0.72 [0.30;2.38] | |
| Maternal age group (years) | | | | | 0.795 | | |
| | 13 -17 | 27 (8.23%) | 23 (7.93%) | 4 (10.5%) | | 1 | - |
| | 18-29 | 210 (64.0%) | 186 (64.1%) | 24 (63.2%) | | 0.59 [0.23;2.05] | 0.596 |
| | 30 - 45 | 91 (27.7%) | 81 (27.9%) | 10 (26.3%) | | 0.56 [0.20;2.22] | 0.589 |
| Gravidity | | | | | 0.845 | | 0.687 |
| | Multigravidae | 238 (73.7%) | 211 (74.0%) | 27 (71.1%) | | 1 | - |
| | Primigravidae | 85 (26.3%) | 74 (26.0%) | 11 (28.9%) | | 1.11 [0.57;2.48] | |
| Parity | | | | | **0.039** | | **0.030** |
| | Multiparity | 164 (70.7%) | 150 (73.2%) | 14 (51.9%) | | 1 | - |
| | Primiparity | 68 (29.3%) | 55 (26.8%) | 13 (48.1%) | | 2.32 [1.13;5.63] | |
| Maternal Hemoglobin | | | | | 0.997 | | 0.854 |
| | HB < 11 | 184 (67.2%) | 162 (66.9%) | 22 (68.8%) | | 1 | - |
| | HB >= 11 | 90 (32.8%) | 80 (33.1%) | 10 (31.2%) | | 0.87 [0.43;2.05] | |
| *S. haematobium* | | | | | 1.000 | | 0.996 |
| | Non-infected | 198 (65.6%) | 175 (65.5%) | 23 (65.7%) | | 1 | - |
| | Positive | 104 (34.4%) | 92 (34.5%) | 12 (34.3%) | | 0.94 [0.49;2.10] | |
| STH | | | | | 0.769 | | 0.806 |
| | Non-infected | 198 (86.5%) | 175 (86.6%) | 23 (85.2%) | | 1 | - |
| | Positive | 31 (13.5%) | 27 (13.4%) | 4 (14.8%) | | 1.04 [0.41;3.62] | |
| Microfilaria | | | | | 0.754 | | 0.665 |
| | Non-infected | 198 (87.6%) | 175 (87.9%) | 23 (85.2%) | | 1 | - |
| | Positive | 28 (12.4%) | 24 (12.1%) | 4 (14.8%) | | 1.17 [0.46;4.09] | |

Data are presented as Odd ratios (OR) and 95% confidence intervals for VitD, P value significance < 0,05, blood for serum parameters was taken from maternal peripheral vein blood or from cord blood, respectively, missing data for each variable were not taken into analysis

**Suppl Table 5. Effects of maternal and/or newborn biometric parameters on cord VitD level:** Data are presented as Odd ratios (OR) and 95% confidence intervals for VitD, *P* value significative < 0,05, blood for serum parameters was taken from maternal peripheral vein blood or from cord blood, respectively, missing data for each variable were not taken into the analysis.

Suppl Table 6: Association of cord $Ca^{2+}$ level with maternal and newborn characteristics

| Variable | | Total | Normal | Abnormal | p. overall | OR [95%CI] | p. ratio |
|---|---|---|---|---|---|---|---|
| Gender | | | | | 0.868 | | |
| | Female | 150 (46.0%) | 132 (45.7%) | 18 (48.6%) | | 1 | |
| | Male | 176 (54.0%) | 157 (54.3%) | 19 (51.4%) | | 0.84 [0.45;1.74] | 0.735 |
| Birth weight (g) | | | | | 0.661 | | |
| | Low [< 2500g] | 14 (4.59%) | 12 (4.43%) | 2 (5.88%) | | 1 | |
| | Normal [>= 2500g] | 291 (95.4%) | 259 (95.6%) | 32 (94.1%) | | 0.49 [0.15;2.55] | 0.673 |
| Birth length (cm) | | | | | 0.649 | | |
| | Abnormal [<45; >54cm] | 13 (4.11%) | 11 (3.93%) | 2 (5.56%) | | 1 | |
| | Normal[45-54cm] | 303 (95.9%) | 269 (96.1%) | 34 (94.4%) | | 0.46 [0.14;2.42] | 0.623 |
| Head circumference (cm) | | | | | 1.000 | | |
| | Abnormal [<30; >38cm] | 12 (3.75%) | 11 (3.86%) | 1 (2.86%) | | 1 | |
| | Normal[30-38cm] | 308 (96.2%) | 274 (96.1%) | 34 (97.1%) | | 0.68 [0.17;5.48] | 0.857 |
| Gestational age (weeks) | | | | | 0.786 | | |
| | Normal [>= 37 weeks] | 275 (87.0%) | 246 (87.2%) | 29 (85.3%) | | 1 | |
| | Preterm [< 37 weeks] | 41 (13.0%) | 36 (12.8%) | 5 (14.7%) | | 1.11 [0.48;3.34] | 0.727 |
| Maternal age group (years) | | | | | 0.169 | | |
| | 13 -17 | 27 (8.23%) | 22 (7.56%) | 5 (13.5%) | | 1 | |
| | 18-29 | 210 (64.0%) | 191 (65.6%) | 19 (51.4%) | | 0.36 [0.15;1.18] | 0.158 |
| | 30 - 45 | 91 (27.7%) | 78 (26.8%) | 13 (35.1%) | | 0.60 [0.23;2.11] | 0.591 |
| Gravidity | | | | | 0.992 | | |
| | Multigravidae | 238 (73.7%) | 212 (73.9%) | 26 (72.2%) | | 1 | |
| | Primigravidae | 85 (26.3%) | 75 (26.1%) | 10 (27.8%) | | 1.03 [0.52;2.39] | 0.819 |
| Parity | | | | | 0.312 | | |
| | Multiparity | 164 (70.7%) | 149 (72.0%) | 15 (60.0%) | | 1 | |
| | Primiparity | 68 (29.3%) | 58 (28.0%) | 10 (40.0%) | | 1.58 [0.75;4.01] | 0.229 |
| Maternal Hemoglobin | | | | | 0.498 | | |
| | HB < 11 | 184 (67.2%) | 166 (68.0%) | 18 (60.0%) | | 1 | |
| | HB >= 11 | 90 (32.8%) | 78 (32.0%) | 12 (40.0%) | | 1.33 [0.67;3.08] | 0.384 |
| *S. haematobium* | | | | | 0.321 | | |
| | Non-infected | 198 (65.6%) | 174 (64.4%) | 24 (75.0%) | | 1 | |
| | Positive | 104 (34.4%) | 96 (35.6%) | 8 (25.0%) | | 0.57 [0.28;1.42] | 0.241 |
| STH | | | | | | | |
| | Non-infected | 198 (86.5%) | 174 (85.7%) | 24 (92.3%) | 0.544 | 1 | |
| | Positive | 31 (13.5%) | 29 (14.3%) | 2 (7.69%) | | 0.46 [0.16;2.35] | 0.382 |
| Microfilaria | | | | | | | |
| | Non-infected | 198 (87.6%) | 174 (89.7%) | 24 (75.0%) | **0.037** | 1 | |
| | Positive | 28 (12.4%) | 20 (10.3%) | 8 (25.0%) | | 2.65 [1.20;7.30] | 0.034 |

Data are presented as Odd ratios (OR) and 95% confidence intervals for $Ca^{2+}$, *P* value significance < 0,05, blood for serum parameters was taken from maternal peripheral vein blood or from cord blood, respectively, missing data for each variable were not taken into analysis

**Suppl Table 6. Effects of maternal and/or newborn biometric parameters on cord $Ca^{2+}$ level:** Data are presented as Odd ratios (OR) and 95% confidence intervals for $Ca^{2+}$, *P* value significative <

0,05, blood for serum parameters was taken from maternal peripheral vein blood or from cord blood, respectively, missing data for each variable were not taken into the analysis.

Suppl Table 7A: Maternal and cord blood VitD levels according to maternal supplementation during pregnancy

| **Variable** | | **VitD supplement N = 228** | **No VitD supplement N = 144** | **p-value** |
|---|---|---|---|---|
| Maternal Blood | | | | |
| | Insufficient (10-29 ng/ml) | 59 (25.9%) | 44 (30.6%) | 0.343 |
| | Sufficient (30 -100 ng/ml) | 169 (74.1%) | 100 (69.4%) | - |
| | Median (IQR) | 33.9 (29.9 to 39.7) | 34.3 (28.6 to 40.5) | 0.927 |
| Cord Blood | | | | |
| | Deficient (< 10 ng/ml) | 3 (1.3%) | 2 (1.4%) | 0.844 |
| | Insufficient (10-29 ng/ml) | 18 (7.9%) | 14 (9.7%) | - |
| | Sufficient (30 -100 ng/ml) | 177 (77.6%) | 108 (75.0%) | - |
| | Toxic (> 100 ng/ml) | 1 (0.4%) | 2 (1.4%) | - |
| | Median (IQR) | 38.8 (34.2 to 45.5) | 41.1 (33.7 to 47.7) | 0.184 |

Data are presented as Odd ratios (OR) and 95% confidence intervals for VitD, *P* value significative < 0,05, blood for serum parameters was taken from maternal peripheral vein blood or from cord blood, respectively, missing data for each variable were not taken into analysis

**Suppl Table 7A: Maternal and Cord Blood VitD D level according to maternal supplementation in pregnancy:** Data are presented as Odd ratios (OR) and 95% confidence intervals for VitD, *P* value significative < 0,05, blood for serum parameters was taken from maternal peripheral vein blood or from cord blood, respectively, missing data for each variable were not taken into analysis.

Suppl Table 7B: Median levels of maternal VitD compared to cord VitD

| **Variable** | | **Maternal VitD** | **Cord VitD** | **p-value** |
|---|---|---|---|---|
| Vitamin D supplement | Median (IQR) | 33.9 (29.9 to 39.7) | 38.8 (34.2 to 45.5) | <0.001 |
| No vitamin D supplement | Median (IQR) | 34.3 (28.6 to 40.5) | 41.1 (33.7 to 47.7) | <0.001 |

Data are presented as median with IQR for VitD, *P* value significative < 0,05, blood for serum parameters was taken from maternal peripheral vein blood or from cord blood, respectively, missing data for each variable were not taken into analysis

**Suppl Table 7B: Maternal VitD compare to Cord VitD***:* Data are presented as median with IQR for VitD, P value significative < 0,05, blood for serum parameters was taken from maternal peripheral vein blood or from cord blood, respectively, missing data for each variable were not taken into analysis.

Suppl Table 8A: Maternal and cord blood calcium levels according to maternal supplementation during pregnancy

| Variable | | Calcium supplement N = 228 | No calcium supplement N = 144 | p-value |
|---|---|---|---|---|
| Maternal Blood | | | | |
| | Insufficient (< 2.2 mmol/l) | 31 (13.6%) | 23 (16.0%) | 0.742 |
| | Sufficient (2.2-2.6 mmol/l) | 174 (76.3%) | 109 (75.7%) | - |
| | Toxic (> 2.6 mmol/l) | 23 (10.1%) | 12 (8.3%) | - |
| | Median (IQR) | 2.3 (2.2 to 2.4) | 2.3 (2.2 to 2.4) | 0.208 |
| Cord Blood | | | | |
| | Insufficient (< 2.2 mmol/l) | 3 (1.3%) | 2 (1.4%) | 0.974 |
| | Sufficient (2.2-3 mmol/l) | 178 (78.1%) | 111 (77.1%) | - |
| | Toxic (> 3 mmol/l) | 18 (7.9%) | 13 (9.0%) | - |
| | Median (IQR) | 2.7 (2.6 to 2.8) | 2.7 (2.6 to 2.8) | 0.755 |

Data are presented as Odd ratios (OR) and 95% confidence intervals for $Ca^{2+}$, *P* value significative < 0,05, blood for serum parameters was taken from maternal peripheral vein blood or from cord blood, respectively, missing data for each variable were not taken into analysis

**Suppl Table 8A: Maternal and Cord Blood Calcium level according to maternal supplementation in pregnancy:** Data are presented as Odd ratios (OR) and 95% confidence intervals for $Ca^{2+}$, P value significative < 0,05, blood for serum parameters was taken from maternal peripheral vein blood or from cord blood, respectively, missing data for each variable were not taken into analysis.

Suppl Table 8B: Median levels of maternal calcium compared to cord calcium

| Variable | | Maternal calcium | Cord calcium | p-value |
|---|---|---|---|---|
| Calcium supplement | Median (IQR) | 2.3 (2.2 to 2.4) | 2.7 (2.6 to 2.8) | <0.001 |
| No Calcium supplement | Median (IQR) | 2.3 (2.2 to 2.4) | 2.7 (2.6 to 2.8) | <0.001 |

Data are presented as median with IQR for $Ca^{2+}$, *P* value significative < 0,05, blood for serum parameters was taken from maternal peripheral vein blood or from cord blood, respectively, missing data for each variable were not taken into analysis

**Suppl Table 8B: Maternal calcium compare to Cord:** Data are presented as median with IQR for $Ca^{2+}$, *P* value significative < 0,05, blood for serum parameters was taken from maternal peripheral vein blood or from cord blood, respectively, missing data for each variable were not taken into analysis.

Suppl Table 9: Serum CRP level according to gravidity and parity

| | | Maternal CRP at birth (N = 375) | | | Cord CRP (N = 328) | |
|---|---|---|---|---|---|---|
| **Variable** | | **Normal[<1mg/dl]** | **Moderate [1-10 mg/dl]** | **Higher [>10 mg/dl]** | **Normal[<1mg/dl]** | **Moderate [1-10 mg/dl]** |
| Gravidity | | | | | | |
| | Primigravidae | 36 (22.6%) | 50 (27.8%) | 7 (23.3%) | 80 (26.0%) | 5 (33.3%) |
| | Multigravidae | 123 (77.4%) | 130 (72.2%) | 23 (76.7%) | 228 (74.0%) | 10 (66.7%) |
| Parity | | | | | | |
| | Primiparity | 32 (27.1%) | 37 (29.1%) | 7 (33.3%) | 65 (29.3%) | 3 (30.0%) |
| | Multiparity | 86 (72.9%) | 90 (70.9%) | 14 (66.7%) | 157 (70.7%) | 7 (70.0%) |
| Age Group | | | | | | |
| | <18 | 13 (8.0%) | 14 (7.7%) | 2 (6.5%) | 22 (7.0%) | 5 (33.3%) |
| | 18-29 | 102 (63.0%) | 118 (64.8%) | 21 (67.7%) | 204 (65.2%) | 6 (40.0%) |
| | >=30 | 47 (29.0%) | 50 (27.5%) | 8 (25.8%) | 87 (27.8%) | 4 (26.7%) |

Data are presented as values or as proportions (%) where indicated, missing data for each variable were not taken into analysis.

**Suppl Table 9: Serum CRP level according to gravidity and Parity:** Data are presented as values or as proportions (%) where indicated, missing data for each variable were not taken into analysis.

Suppl Table 10: Unadjusted and multivariable logistic regression investigating the association between VitD concentrations with maternal serum CRP levels (N = 375)

| | | Univariable | | | Bivariable | | Multivariable | |
|---|---|---|---|---|---|---|---|---|
| **Variable** | | **CRP<1 (n = 145)** | **CRP>=1 (n = 183)** | **p-value** | **cOR[95%CI]** | **p-value** | **aOR[95%CI]** | **p-value** |
| Maternal VitD | | | | 0.642 | | 0.642 | | 0.342 |
| | Sufficient (30 -100 ng/ml) | 102 (70%) | 133 (73%) | | 1 | | 1 | |
| | Insufficient (10-29 ng/ml) | 43 (30%) | 50 (27%) | | 0.89 [0.55, 1.45] | | 0.74 [0.40, 1.37] | |
| Cord VitD | | | | 0.008 | | | | |
| | Sufficient (30 -100 ng/ml) | 120 (83%) | 167 (91%) | | 1 | | 1 | |
| | Insufficient (10-29 ng/ml) | 21 (14%) | 12 (6.6%) | | 0.41 [0.19, 0.85] | 0.02 | 0.25 [0.08, 0.70] | 0.012 |
| | Deficient (< 10 ng/ml) | 1 (0.7%) | 4 (2.2%) | | 2.87 [0.42, 56.6] | 0.348 | 1.87 [0.23, 39.0] | 0.595 |
| | Toxic (> 100 ng/ml) | 3 (2.1%) | 0 (0%) | | 0 [NA] | 0.985 | 0 [NA] | 0.985 |

Data are presented as values or as proportions (%), core odd ratios (cOR), adjusted odd ratios (aOR), p value < 0,05 and 95% confidence intervals where indicated for VitD and adjusted by age group, parity and *Sh* infection status, blood for serum parameters was taken from maternal peripheral vein blood or from cord blood, respectively, missing data for each variable were not taken into analysis

**Suppl Table 10: Unadjusted and Multivariate logistic regression investigating the association between VitD concentrations with maternal serum CRP levels:** Data are presented as values or as proportions (%), core odd ratios (cOR), adjusted odd ratios (aOR), p value < 0,05 and 95% confidence intervals where indicated for VitD and adjusted by age group, parity and *Sh* infection status, blood for serum parameters was taken from maternal peripheral vein blood or from cord blood, respectively, missing data for each variable were not taken into analysis

Suppl Table 11: Unadjusted and multivariable logistic regression investigating the association between VitD concentrations with maternal eosinophils (N = 375)

| | | Univariable | | | Bivariable | | Multivariable | |
|---|---|---|---|---|---|---|---|---|
| **Variable** | | **Normal** | **Abnormal** | **p-value** | **cOR[95%CI]** | **p-value** | **aOR[95%CI]** | **p-value** |
| Maternal VitD | | | | 1 | | 0.935 | | 0.056 |
| | Sufficient (30 -100 ng/ml) | 168 (70%) | 9 (69%) | | 1 | | 1 | |
| | Insufficient (10-29 ng/ml) | 71 (30%) | 4 (31%) | | 1.05 [0.28, 3.34] | | 5.72 [1.03, 44.4] | |
| Cord VitD | | | | 0.342 | | | | |
| | Sufficient (30 -100 ng/ml) | 207 (87%) | 11 (85%) | | 1 | | 1 | |
| | Insufficient (10-29 ng/ml) | 26 (11%) | 1 (7.7%) | | 0.72 [0.04, 3.96] | 0.761 | 2.41 [0.12, 18.6] | 0.453 |
| | Deficient (< 10 ng/ml) | 4 (1.7%) | 1 (7.7%) | | 4.7 [0.23, 35.4] | 0.182 | 18.66 [0.00, Inf] | 0.057 |
| | Toxic (> 100 ng/ml) | 2 (0.8%) | 0 (0%) | | 0 [NA] | 0.994 | 0 [NA] | 0.996 |

Eosinophils data are presented as values or as proportions (%), core odd ratios (cOR), adjusted odd ratios (aOR), 95% confidence intervals and p value < 0,05 where indicated, Neutrophils is Normal if Eosinophils abs: [0.0 - 1.3]; missing data for each variable were not taken into analysis.

**Suppl Table 11: Unadjusted and Multivariate logistic regression investigating the association between VitD concentrations with maternal Eosinophils :** Eosinophils data are presented as values or as proportions (%), core odd ratios (cOR), adjusted odd ratios (aOR), 95% confidence intervals and p value < 0,05 where indicated, Neutrophils is Normal if Eosinophils abs: [0.0 - 1.3]; missing data for each variable were not taken into analysis.
